# What’s in a loop?

**DOI:** 10.1186/1478-811X-10-31

**Published:** 2012-10-30

**Authors:** Stephan M Feller, Marc Lewitzky

**Affiliations:** 1 Biological Systems Architecture Group, Weatherall Institute of Molecular Medicine, University of Oxford, Oxford, OX3 9DS, UK

## Abstract

DNAs and proteins are major classes of biomolecules that differ in many aspects. However, a considerable number of their members also share a common architectural feature that enables the assembly of multi-protein complexes and thereby permits the effective processing of signals: loop structures of substantial sizes. Here we briefly review a few representative examples and suggest a functional classification of different types of loop structures. In proteins, these loops occur in protein regions classified as intrinsically disordered. Studying such loops, their binders and their interactions with other loops should reveal much about cellular information computation and signaling network architectures. It is also expected to provide critical information for synthetic biologists and bioengineers.

## 

DNAs are different from proteins in many ways. Our genomic DNA molecules are vast, even when compared to the largest proteins we know. DNA is essentially composed of 4 building blocks that are at best modified with a few extra side bits here and there. In proteins we find at least 20 different amino acids and more than one hundred types of posttranslational modifications.

The genomic DNAs of eukaryotes live mostly in one confined cell compartment, while proteins lurk in virtually every corner of the cell and many of them whiz about.

DNA seems to be usually just able to coil into spirals that coil into bigger spirals (30 nm fibre) that coil into even bigger spirals (200 nm fibre/chromosome), while proteins can take up a plethora of diverse and highly complex shapes, or, for intrinsically disordered proteins, no apparent shapes at all.

Most DNA seems to have the capacity to live forever, while probably all proteins have a quite limited lifespan.

Despite all of these differences, DNA and proteins have of course a number of things in common. Both are extremely important classes of biomolecules and both are, for example, able to store information. In addition, they share an architectural feature related to complex information processing: substantially sized loop structures.

In genomic DNAs, these loops have already been studied for decades and in some detail, but exciting new results are still constantly emerging [1-3].

To reveal its information, DNA must be untangled and often distant regions within one molecule or between fellow DNA molecules have to interact. These communicating loops enable promoters, enhancers and other regulatory elements, which are sometimes megabases apart, to come together in space and time in a highly dynamic process which is not entirely understood [4,5]. An early example for this type of long-distance interaction was the finding that the beta-globin enhancer, which is located far upstream of the globin genes, comes into close proximity when the genes are actively transcribed [6]. New methodologies, for example the Hi-C method [7-9], are now addressing DNA looping at the whole genome level. Here, protein-DNA complexes at interacting loci are preserved by fixation with formaldehyde, affinity purified and subsequently analyzed by high-throughput sequencing [9].

Apart from their crucial participation in information transfer, DNA loops also play an important role in DNA maintenance. Loop structures at the telomeric ends of chromosomes safeguard and prevent these ends from being treated as DNA double-strand breaks [10]. When the telomeric ends become critically short, loop structures are absent which eventually will result in cell cycle arrest [11].

It goes without saying that loops also play many critical roles in RNA molecules, although they are, to our knowledge, usually not as directly involved in signal processing by protein complex cross-talk.

Proteins use loops too, and in a gamut of contexts. Loop regions occur in inter-domain segments of otherwise well-folded proteins, where they can serve multiple functions: short loops sometimes feature as mere linkers or may also provide the required flexibility for the movement of the neighboring protein domains (linker loops [L-L]). Other loops serve as linkers regions, but also allow proteins to interact intramolecularly when undergoing shape changes (intramolecular docking loops [IMD-L]). The linker regions between the SH2s and catalytic domains of Src and Abl kinases [12,13] (and references therein) and the linker region around tyrosine 221, between the SH3 domains of the human c-Crk II protein, are well-studied examples proven to be essential for intramolecular protein binding events [14,15] (and Figure 1). Then there exists a vast number of loop regions which upon modification by specific enzymes serve as docking sites for a single protein interaction partner, or a couple of them (small docking loops [SD-L]). Such loops are found, for example, between the membrane-spanning helices of receptor and channel proteins that reside in cellular membranes. Short loops localized within a well-folded protein domain can also work together to form binding pockets for proteins and a range of other biomolecules (binding pocket loops [BP-L]).

**Figure 1:**
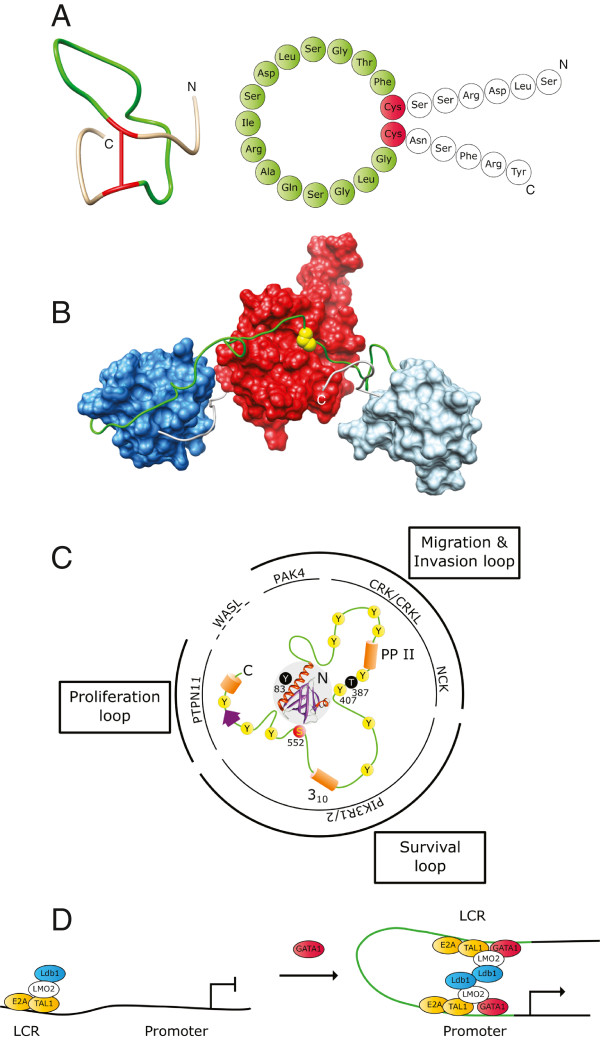
**Selected examples for different functional types of loops in large biomolecules. A**. Atrial natriuretic peptide hormones contain vital ‘activity conferring’ loops (green) generated in each case by a single disulfide bond (red). Shown here is the solution conformation and cartoon representation of one ANP variant. Solution conformation generated from PDB database entry 1ANP [16] using Chimera [17]. Cartoon representation based on similar representation in [18]. **B**. The ‘intramolecular docking’ loop (green) in the c-Crk II protein regulates the overall conformation of its SH2 (red), SH3N (dark blue) and SH3C (light blue) domains by an inducible intramolecular interaction between the SH2 domain and a phosphorylated tyrosine residue (yellow) in the loop region. Structural representation generated from PDB entry 2DVJ (aa 1-228; SH2, SH3N and loop) and PDB entry 2EYZ (aa 229-304; loop and SH3C) [15]. **C**. Gab1 contains an N-terminal PH domain (grey shaded area) followed by a largely unstructured region (green) with numerous sites for potential intra- and intermolecular interactions (yellow: tyrosine-phosphorylation sites, red: serine-phosphorylation site, orange and purple: secondary structure elements). This ‘signal computation’ loop permits the assembly of and signaling via context-specific complexes [19,20]. The poly-proline type II helix (PP II) and the 3_10_ helix (3_10_) in Gab1 and its close relative Gab2 can interact with the Grb2 adapter protein [21,22]. Cortactin was also reported to interact with these regions [23]. pTyr407 was mapped as binding site for NCK [24]. pSer 552 allows intramolecular interaction of the loop region with the PH domain and regulates Gab1 localization [25]. Interaction regions for PAK4 kinase [26], CRK-family proteins [27,28], PI3 kinase (PIK3R1/2; [29]) and the phosphatase SHP2 (PTPN11; [30]) have been described. WASL (N-WASP) may interact directly with the PH domain (Richard Vaillancourt, Morag Park et al.; personal communication). Elements associated with specific cellular functions like cell motility, survival or proliferation are often found co-localized in defined regions of the signaling loop. Y83 and T387 are two residues mutated in breast cancer. The Y83C mutation could interfere with PI(3,4,5)P3 binding of the PH domain [25], while a T387N mutation abolishes a threonine residue phosphorylated after EGFR or c-Met stimulation [31]. Structural representation of PH domain generated from PDB entry 2X18. **D**. A ‘signal computation’ loop (green) in a DNA molecule controls transcriptional activity by bringing locus control region (LCR) and promoter region together in the presence of a crucial transcription factor (red: GATA1). Based on a similar figure in [1] .

In extracellular proteins and polypeptides, functionally vital loop structures, for example generated by disulfide bonds, are found in a vast range of contexts. Classical examples are the loops of the atrial natriuretic peptide hormone family members [32] (and Figure 1). These loops could be designated ‘activity conferring loops’ [AC-L].

Finally, the human proteome encompasses many proteins suspected to contain much larger loops with numerous putative sites for protein docking [33]. Such larger loops are thought to assemble crucial parts of molecular ‘nanocomputers’, which compute signaling input from environment-sensing transmembrane receptors [19,20,33] (and Figure 1). These could be designated as ‘signal computation loops’ [SC-L]). This type of loops is quite reminiscent of their DNA counterparts, which are, amongst other things, involved in transcriptional regulation. In proteins, they appear preferentially in the ‘anarchic fraction’ of proteomes; in humans approximately one third of the proteome is thought to consist of partially or mostly ‘unstructured’ i.e. ‘intrinsically disordered’ proteins.

These fickle and certainly understudied characters lack in many sections of their protein chains secondary and tertiary structure elements that would be detectable with the most commonly used current structure prediction programs. Some of those proteins display disordered regions of several to many hundred amino acids, which made it for a long time difficult to understand the molecular mechanisms of how they actually conduct their business in cells. A long and unrestrained amino acid chain that is able to move about freely can in principle adopt a virtually infinite number of conformations. So, presumably, none of these large molecules would ever take on an identical shape. Yet they do perform their usually complex duties in cells swiftly and effectively.

Protein chain loops are, of course, a feature that can make this possible [19]. Looping will substantially restrict the conformational space that protein chains can sample, but at the same time leave enough flexibility to allow multiple other proteins to bind and covalently or noncovalently modify the intrinsically disordered protein chains. Importantly, protein loop structures of sufficient size should easily be able to simultaneously interact with several other protein and/or DNA loops, thereby enabling diverse and complex signal computation operations through the cross-talk of two or more signaling pathways in a simple yet elegant manner.

At present, we still know relatively little details about large protein loops involved in signal processing. Nevertheless, it would seem to be a fair guess that the unstructured parts of many proteins ‘live’ in a zone of intermediate flexibility between the hypothetical structural poles of ‘complete chaos’ on one end and substantial rigidity imposed through extensive constraints by a highly folded protein on the other end.

That is not to say that all disordered proteins must use loops. Some proteins and in particular those that lack any well-folded regions and are relatively short may rely entirely on a more or less linear protein chain for their biological actions and, for example, adopt local structure only upon interaction with their binding partner(s). The binding of p27Kip1 to the Cdk2/cyclinB complex is a well-known point in case [34].

Nevertheless, higher order signal processing events can be expected to rely in many cases on two or more loops structures that enable the coordinated assembly of sub-complexes, which can then communicate to decide cell fates and orchestrate and direct the subsequently required cell actions. In order to generate and organize these loop structures in intrinsically disordered protein chains an ‘organizing center’ is probably mandatory. In many proteins, for example those of the Gab, Irs/Dok, Frs and p130Cas/BCAR1 families, well-folded N-terminal domains might serve as anchor points [33,35,36]. In principle, such an organizing center could also be more C-terminally located, or there could be more than one such center within a single protein chain.

So what about large disordered proteins without a recognizable folded region? It is conceivable that these characters could use other biomolecules, most commonly other proteins, as helpers to ‘get into shape’. Stabilizing loops that are built around a folded domain could in some cases also be supported by ‘loop-stabilizing intermolecular interactions in the form of homo- and hetero-oligomers. Proteomic studies of such native complexes under gentle conditions using ion mobility mass spectrometry [37] might provide first insights into the key components involved and into some details of their interactions, hence providing a basis for further detailed biophysical analyses.

We have learned a lot about the individual building blocks in cells and their potential interaction partners in recent years. Now we need to put the pieces together, to develop a much better architectural understanding of the molecular processes in cells. A functional classification of loop structures, such as we have suggested here, might help along the way, although it is clear that nature’s ingenuity and use of highly versatile molecular themes that are almost endlessly varied will finally defeat our brains’ apparent urge to put everything we encounter into neat little boxes.

An architectural, more holistic view of the highly dynamic living cells can, of course, be only obtained by combining a wide range of methodologies, including different super-resolution microscopy techniques [38-41], in-cell NMR [42-46], high speed in solution atomic force microscopy [47] and cryoEM [48-51], to name but a few. Studying proteins, as much as possible, in their natural habitat [52] will also substantially contribute to a more life-like model of cell architectures and cell signaling networks.

In addition, we probably need new ways to identify, describe and classify the highly dynamic, often disordered regions and arrangements of proteins, for example along the lines suggested by Peter Tompa [53] and others [54-56]. Accurate descriptions of loop structures may not be entirely trivial to obtain, but there are several algorithms and databases readily available to model loops in proteins [57-59]. We would like to argue, that for a full description, biophysical classifications should be combined with functional descriptions, such as the one proposed in an earlier section of this manuscript.

These are exciting times for cell biologists, biochemists, biophysicists and molecular biologists, but also for chemists, physicists and engineers. As more and more research fields interconnect to answer together questions that seemed far out of reach just a few years back, we can expect to soon reach new shores on the fascinating journey into biological nanospace. The conceptual trinkets and architectural maps we will bring back from these travels should provide vital clues for better treatments of diseases, synthetic biology and a wide range of bioengineering tasks.
